# Red-light photobiomodulation improves cognition and neuropsychiatric symptoms in post-stroke cognitive impairment: a randomized trial

**DOI:** 10.3389/fneur.2025.1634701

**Published:** 2025-11-26

**Authors:** Xuerong Huang, Zihui Sun, Weijie Wu, Linli Lou, Panpan Wang, Qin Wang, Yuanyu Fu, Liange Xu, Mengwan Song, Lidong Zhu, Jin Huang, Shaobo Ni, Guangyong Chen, Xueping Liu, Zhiqian Tong

**Affiliations:** 1 The Third Affiliated Hospital, Wenzhou Medical University, Wenzhou, Zhejiang, China; 2 Department of Geriatric Neurology, Shandong Provincial Hospital, Cheeloo College of Medicine, Shandong University, Jinan, Shandong, China; 3 Provincial Hospital Affiliated to Shandong First Medical University, Jinan, Shandong, China; 4 Department of Science and Education, Beijing Geriatric Hospital, Beijing, China; 5 Zhejiang Provincial Clinical Research Center for Mental Disorders, The Affiliated Wenzhou Kangning Hospital, School of Mental Health, Wenzhou Medical University, Wenzhou, Zhejiang, China

**Keywords:** formaldehyde, red-light therapy, randomized controlled trial, post-stroke cognitive impairment (PSCI), stroke

## Abstract

**Introduction:**

Acute stroke patients often develop post-stroke cognitive impairment (PSCI) and dysthymic disorders. Formaldehyde (FA) induces cognitive decline and depression; while red light (RL) at 630 nm can degrade FA by activating FA-dehydrogenase (FDH). This study investigates the therapeutic effects of a novel RL device on cognitive function and neuropsychiatric symptoms in patients with PSCI.

**Methods:**

This was an exploratory, parallel-group, randomized controlled trial with concealed allocation, assessor blinding and intention-to-treat analysis. Stroke patients (*n* = 90) were enrolled. A total of 38 patients in the PSCI group and 44 patients in the PSCI-RL group completed the study. Participants were followed for 6 months, during which the intervention phase comprised 3 months of RL therapy or sham stimulation, followed by continued follow-up. Cognitive [Montreal Cognitive Assessment (MoCA)/Mini-Mental State Examination (MMSE)], neuropsychiatric [Hamilton Depression Rating Scale (HAMD), Hamilton Anxiety Rating Scale (HAMA)], and functional [Barthel Index (BI)] assessments were conducted at baseline and 6 months. Blood and urine levels of key enzymes and metabolites involved in FA metabolism were quantified, including semicarbazide-sensitive amine oxidase (SSAO, an FA-generating enzyme), FDH (an FA-degrading enzyme), cytochrome c (Cyt-c), FA, hydrogen peroxide (H_2_O_2_, a marker of FA generation), and coenzyme Q10 (CoQ10, an endogenous FA scavenger).

**Results:**

The phototherapy device improved cognitive abilities, reduced anxiety and depression, lessened stroke severity, and enhanced daily living activities in the PSCI-RL group at 6 months. Additionally, RL therapy altered FA metabolism, as it lowered SSAO and H_2_O_2_ levels and increased FDH, Cyt-c, and CoQ10 in blood and/or urine of PSCI patients.

**Conclusions:**

RL therapy may improve clinical symptoms in post-stroke patients by modulating FA metabolism, suggesting a safe and promising approach for treatment and rehabilitation.

**Clinical trial registration:**

https://www.chictr.org.cn/showproj.html?proj=159956, Identifier: ChiCTR2200058991.

## 1 Introduction

With the increase of the aging population, the incidence of stroke is also increasing. Stroke is one of the world's leading causes of death and disability (1, 2). This means a huge financial burden on families and society (3). Post-stroke cognitive impairment (PSCI) refers to a series of syndromes that meet the diagnostic criteria for cognitive impairment within 6 months of a stroke occurring (4). PSCI spans a clinical spectrum from mild cognitive impairment (MCI) to severe post-stroke dementia (PSD) (5). PSCI is primarily characterized by problems with executive function, memory, attention, language, and visuospatial function (6). PSCI occurs in about 1/3 of patients who experience a stroke (7). Of particular note, a recent follow-up study revealed that PSCI could occur in as many as 61% of patients 10 years after the stroke onset (8). Patients with PSCI progressively worsen and 20%−30% develop PSD (9). PSCI leads to poorer functional prognosis, higher dependency, mortality, and risk of stroke recurrence in patients (10).

Current interventions for PSCI include control of vascular risk factors, pharmacotherapy (11), and various forms of physical stimulation, such as repetitive transcranial magnetic stimulation (rTMS) (12, 13), virtual reality rehabilitation training (14), and phototherapy (PT) or photobiomodulation (PBM) (15). Recent studies have implicated formaldehyde (FA) in cognitive decline (16, 17) and emotional disturbances (18) in both animal models and human subjects. At the mechanistic level, preclinical research has demonstrated that 630-nm red light (RL) therapy can ameliorate cognitive impairment and depressive-like behaviors by activating formaldehyde dehydrogenase (FDH), modulating FA metabolism, and attenuating oxidative stress (19, 20). Given that oxidative stress is a central pathophysiological process in both ischemic and hemorrhagic stroke (21, 22), these findings provide a compelling rationale for investigating RL therapy as a potential intervention for PSCI. However, the current evidence is largely derived from animal experiments and early preclinical studies; its efficacy, safety, and specific mechanisms in humans—particularly in relation to FA metabolic modulation—remain insufficiently substantiated by clinical data.

Therefore, rigorously designed clinical trials are needed to validate the therapeutic efficacy of RL therapy and to elucidate its underlying mechanisms. Against this background, the present study employs 630-nm red light therapy to investigate its therapeutic effects in PSCI patients and to further explore its role in regulating endogenous FA metabolic pathways.

## 2 Method

### 2.1 Subjects

This study was conducted in the Third Hospital of Wenzhou Medical University. A total of 90 patients with PSCI were recruited in this study between September 2022 and June 2024. The study procedures and protocols were approved by the Ethics Committee of the Third Affiliated Hospital of Wenzhou Medical University (Approval No. YJ2022008), and all participants or their guardians signed a written informed consent form. The study was registered on Chictr.org (China Clinical Trial Registry Unique Identifier, registration number: ChiCTR2200058991), date of registration: April 22, 2022.

The inclusion criteria were as follows: (1) diagnosis of acute stroke according to the Chinese Medical Association's diagnostic criteria (23), with stable vital signs and confirmation by a professional neuroradiologist via cranial CT or MRI; (2) pre-onset Modified Rankin Scale (mRS) score of ≤ 2 points and no history of cognitive impairment; (3) ability to cooperate with relevant assessments and tests, and cognitive impairment [defined as a Montreal Cognitive Assessment (MoCA) score < 26] at enrollment; (4) aged 40–80 years old; (5) enrollment within 2 weeks after stroke onset.

The exclusion criteria are as follows: (1) severe hearing or speech impairment; (2) various causes of mental abnormality or epilepsy or inability to properly assess cognitive functioning; (3) persons with cerebral and cardiac pacemakers; history of specialty and multiple drug allergies; (4) those with a clear etiology of cognitive impairment such as syphilis, ehrlichiosis, thyroid dysfunction, VitB12 deficiency, Notch3 gene mutation, etc.; (5) severe functional failure of vital organs and critical condition with a life expectancy of fewer than 6 months; (6) anyone who for any reason is unable to complete therapy, neuropsychological evaluations, neuroimaging, or other examinations; (7) those who are conducting other clinical trials or who, in the judgment of the investigator, have other conditions that may interfere with this study.

Case exclusion criteria: (1) inclusion of those found not to meet the diagnostic criteria for PSCI (4) after inclusion and who were inadvertently included; (2) included cases who have not received the required intervention for various reasons; (3) other treatments not within the scope of the regulation, in particular the combination of other therapeutic measures (including drugs or non-drugs) that have a significant impact on this clinical trial, which affect the judgment of efficacy and safety, and is clearly not in accordance with the protocol or is contrary to the protocol.

### 2.2 Equipment

**Figure 1:**
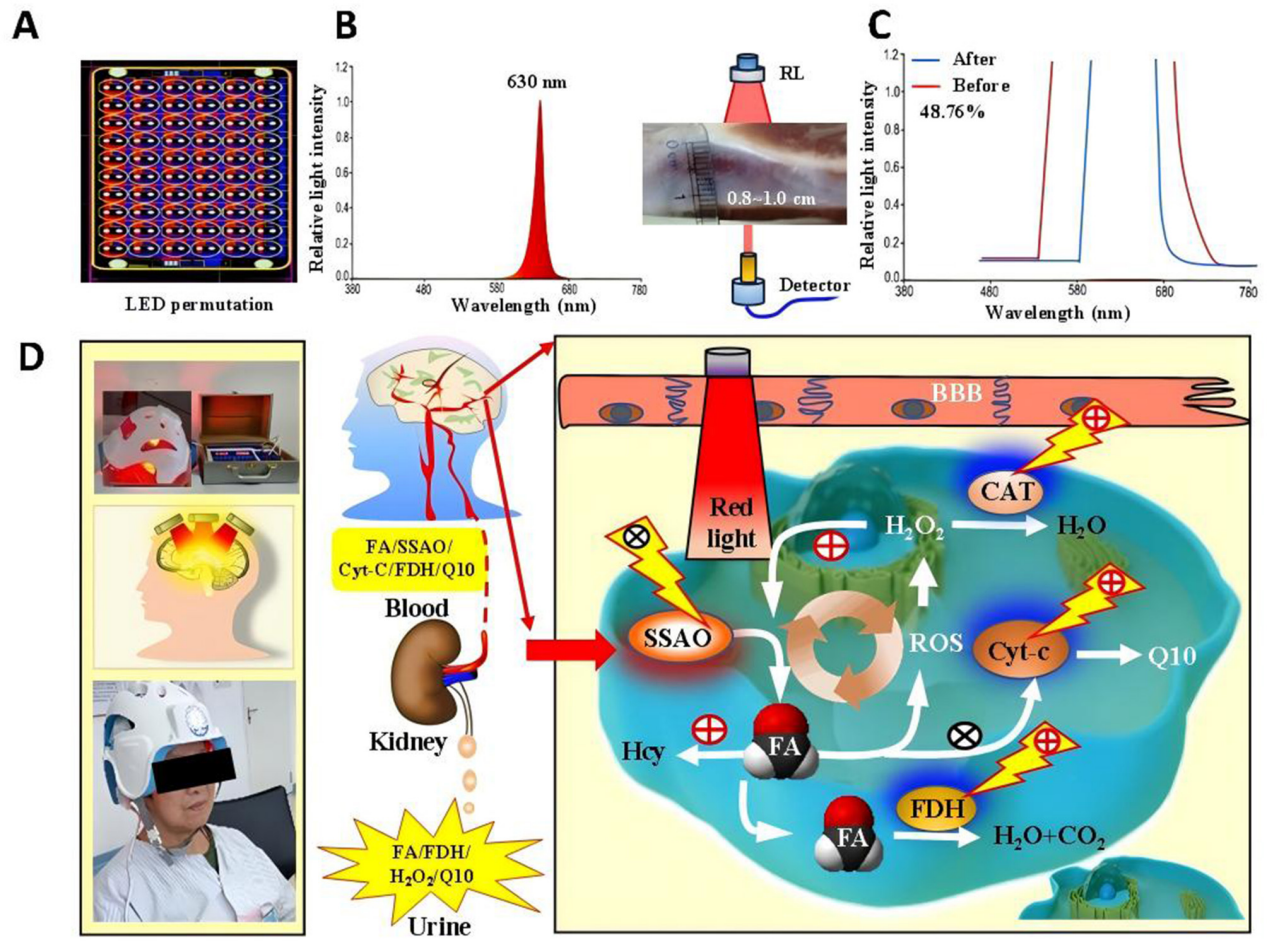
The novel phototherapy device with red light and its effects on FA levels in the blood of PSCI patients **(A–C)**. The penetration rate in this new 630-nm RL device **(D)**.

The High-Energy Red Light Device, invented by Professor Tong Zhiqian from our research group (China Invention Patent: ZL2015103542675), and the novel wearable optoelectronic integrated brain rehabilitation device (China Utility Device Quality Supervision and Inspection Center of the National Medical Products Administration and meets the standards of YY 0505-2012 Medical Electrical Equipment—General Requirements for Safety: Electromagnetic Compatibility and YY 0607-2007 Medical Electrical Equipment—Particular Requirements for the Safety of Nerve and Muscle Stimulators (Figures 1A–D). The new device emitted 630-nm red light from LEDs, which was able to penetrate a skull 0.8–1.0 cm in thickness with an estimated penetration rate of approximately 48% (24) (Figures 1A–C). Then, we examined the changes of FA-related metabolism including: semicarbazide-sensitive amine oxidase (SSAO), FDH, Cyt-c, FA, H_2_O_2_, and CoQ10 in the blood and urine in the PSCI patients with or without RL therapy (Figure 1D). Model Patent: CN 209865048U were used in this study. The device was manufactured by the Beijing Institute of Major Brain Diseases and ZPARK Zhongguancun High-Tech Enterprise, model JGHNY-Q. It has been tested by the Beijing Medical Participant recruitment, assessment, intervention, and follow-up were conducted in the Third Hospital of Wenzhou Medical University. The case report forms were filled out and kept in the Third Affiliated Hospital of Wenzhou Medical University.

### 2.3 Interventions

**Figure 2:**
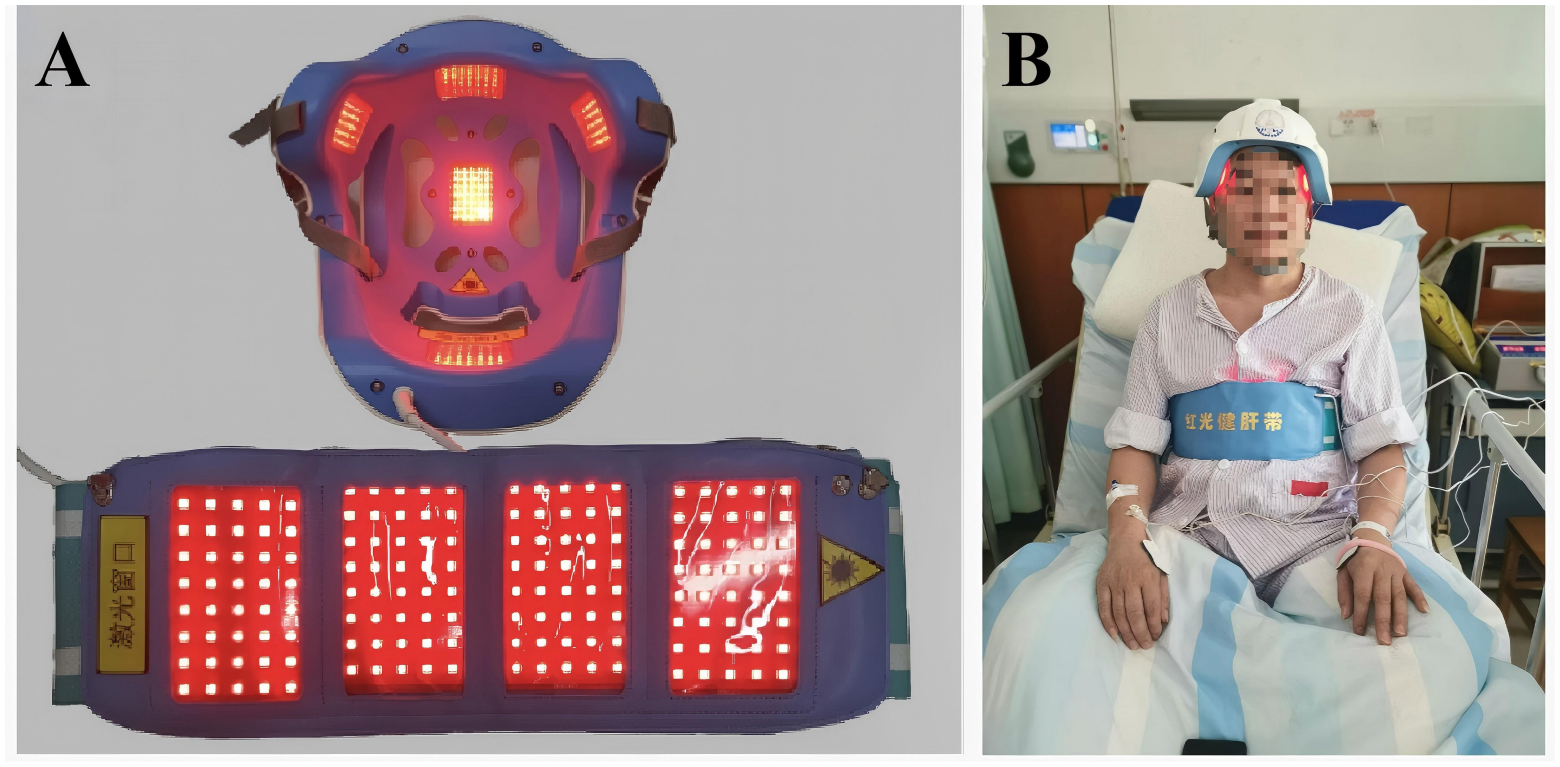
The therapeutic device: a 630-nm red light device used for the treatment of PSCI patients. **(A)** Photographs of the activated therapeutic LED light source employed in the management of PCSI patients. **(B)** Images of patients undergoing treatment while wearing red-light device.

The PSCI-RL group received RL irradiation in addition to conventional treatment for a period of 3 months. Treatment was performed by researchers or caregivers trained in the operation of the wearable brain rehabilitation device. As shown in Figures 1, 2, subjects wore LED helmets, LED belly bands, and electrode pads while receiving transcranial and transabdominal irradiation combined with manual electrotherapy. The irradiation wavelength was 630 ± 15 nm, with each LED emitting 5 mW of power. The power density was 20 mW/cm^2^ over the anterior helmet LEDs, 40 mW/cm^2^ over the posterior helmet LEDs, 5 mW/cm^2^ over temporal helmet LEDs, and 5 mW/cm^2^ over the abdominal LEDs. Each treatment session lasted 30 min, performed five times per week, for a total intervention duration of 3 months. A treatment schedule was developed for each participant in the intervention group to ensure consistency. During the 6-month study period, both groups received identical conventional rehabilitation and manual electrotherapy delivered via electrode pads with active electrical stimulation. The only difference was that the PSCI-RL group underwent simultaneous RL irradiation (630 ± 15 nm) through the LED helmet and abdominal belt, whereas in the PSCI control group the red light devices were applied in the same manner but kept switched off (sham stimulation). This ensured that both groups received equivalent co-interventions in terms of electrical stimulation, with the active RL component being the sole variable between groups.

### 2.4 Clinical assessments

The evaluation of cognitive function and neuropsychiatric symptoms, as well as the capacity to perform activities of daily living (ADL), was conducted for all subjects. Assessments were systematically administered at baseline and 6 months throughout the study period.

#### 2.4.1 MMSE

Mini-Mental State Examination (MMSE) is the most commonly used assessment for measuring cognitive functioning (25). The scale, encompassing a total score of 30 points, evaluates domains such as orientation, memory, attention and calculation, recall, and language. Higher scores on this scale are indicative of superior cognitive functioning. It serves as a valuable tool for monitoring alterations in a patient's cognitive abilities over time.

#### 2.4.2 MoCA

Relative to the MMSE, the MOCA has advantages for screening for mild cognitive impairment, with high sensitivity and specificity (26). Its total score is also 30, involving cognitive domains such as memory, executive function, attention, language, abstraction, naming, delayed recall, and orientation, with higher scores indicating better cognitive function.

#### 2.4.3 HAMA

Emotional states were assessed using the Hamilton Anxiety Rating Scale (HAMA), which was developed by Hamilton in 1959 (27). It includes 14 items, all of which are rated on a 5-point scale from 0 to 4; a total score of more than 29 may indicate severe anxiety; if it is more than 21, there must be significant anxiety; if it is more than 14, there is anxiety; if it is more than 7, there is likely to be anxiety; and if the score is less than 7, there are no symptoms of anxiety (28).

#### 2.4.4 HAMD

There is also the Hamilton Depression Rating Scale (HAMD) which was developed by Hamilton in 1960 (29). The 17-item version, divided into seven factors, is used in this study, with most items on a 5-point scale from 0 to 4, and a few on a 3-point scale from 0 to 2. Total score < 7: normal; total score 7–17: possible depression; total score 17–24: definitely depressed; total score >24: major depression.

#### 2.4.5 NIHSS

The National Institutes of Health Stroke Scale (NIHSS) is a standardized, quantitative tool used to objectively quantify the severity of neurological deficits in acute stroke patients. Developed in 1989 (30), the scale comprises 15 items that evaluate key neurological domains, including level of consciousness, gaze, visual fields, motor and sensory function, ataxia, language, and neglect. The total score ranges from 0 to 42, with a higher score indicating more severe neurological impairment. It is widely employed in clinical trials and practice for its reliability and sensitivity to change.

#### 2.4.6 BI

The Barthel Index (BI) is a validated and widely used ordinal scale for measuring functional performance in basic activities of daily living (ADL). Originally developed by Dorother Barthel and Floorence Mahoney in 1965 (31), it assesses ten essential activities: feeding, bathing, grooming, dressing, bowel control, bladder control, toileting, chair-bed transfers, ambulation on level surfaces, and stair climbing. The total score ranges from 0 to 100, where a higher score denotes greater independence and reduced need for caregiver assistance. Recognized for its simplicity, high inter-rater reliability, and sensitivity, the BI remains a cornerstone instrument in rehabilitation medicine and stroke outcome research.

#### 2.4.7 mRS

The mRS is a widely used measure of global disability, comprising seven grades from 0 to 6 that reflect the level of functional dependence in daily activities (32). Higher scores denote more severe disability and dependence, with 0 representing no symptoms and 6 indicating death.

#### 2.4.8 BPRs

The Brief Psychiatric Rating Scale (BPRS) is a clinician-rated instrument designed to assess the severity of psychopathology across a broad range of symptom domains. Developed by Overall and Gorham in 1962 (33), the 18-item version used in this study captures symptoms such as somatic concern, anxiety, emotional withdrawal, guilt feelings, and unusual thought content. Each item is rated on a 7-point scale (1 = not present to 7 = extremely severe), with the total score providing a global index of psychiatric symptom burden. It is frequently utilized to monitor treatment response in patients with neuropsychiatric comorbidities.

### 2.5 Sample size calculation and random allocation

This study was a randomized controlled trial with red light irradiation plus conventional medication in the treatment group and conventional medication in the control group. The MoCA score of the study subjects was the observed outcome indicator. Based on a preliminary analysis of initially enrolled patients that established the control group's MoCA baseline at 12.12 ± 5.01 points, and drawing on effect sizes reported in prior effective interventions for PSCI (34), we set the expected MoCA improvement in the RL treatment group at 4.2 points for sample size calculation. Using PASS 2021 software, the sample size of the PSCI-RL group was calculated to be N1 = 31 cases and the sample size of the PSCI group was calculated to be N2 = 31 cases, taking into account the loss of visits as well as the refusal of visits at 30%, the final minimum number of subjects needed for the PSCI-RL group and the PSCI group was 45 cases each, for a total of a minimum of 90 subjects to be included in the study. The formula is calculated as follows (35):


$$\begin{array}{l} n=\frac{{\left(Z_{\alpha }+Z_{\beta }\right)}^{2}*\;{2\sigma }^{2}}{\delta^{2}} \end{array}$$

**Figure 3:**
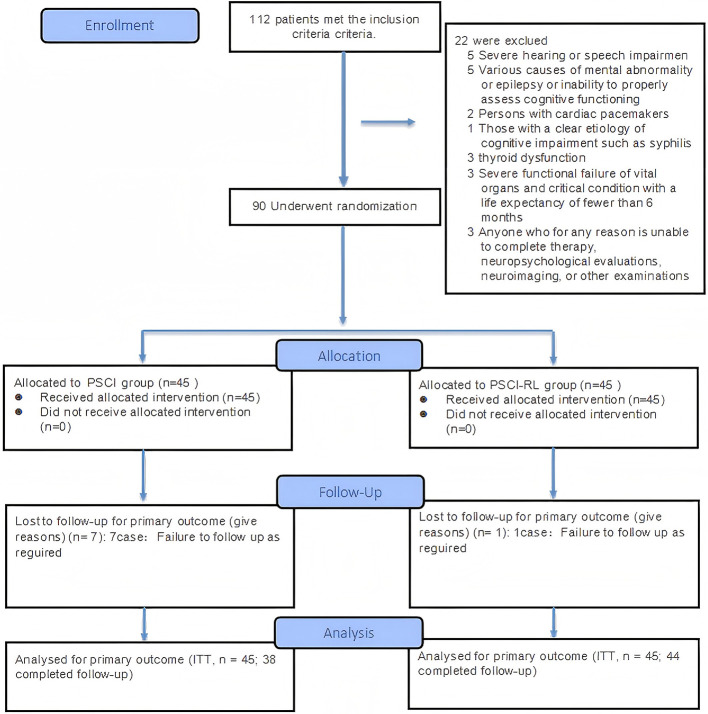
CONSORT 2025 flow diagram of participant progression through enrollment, allocation, follow-up, and analysis phases.

**Figure 4:**
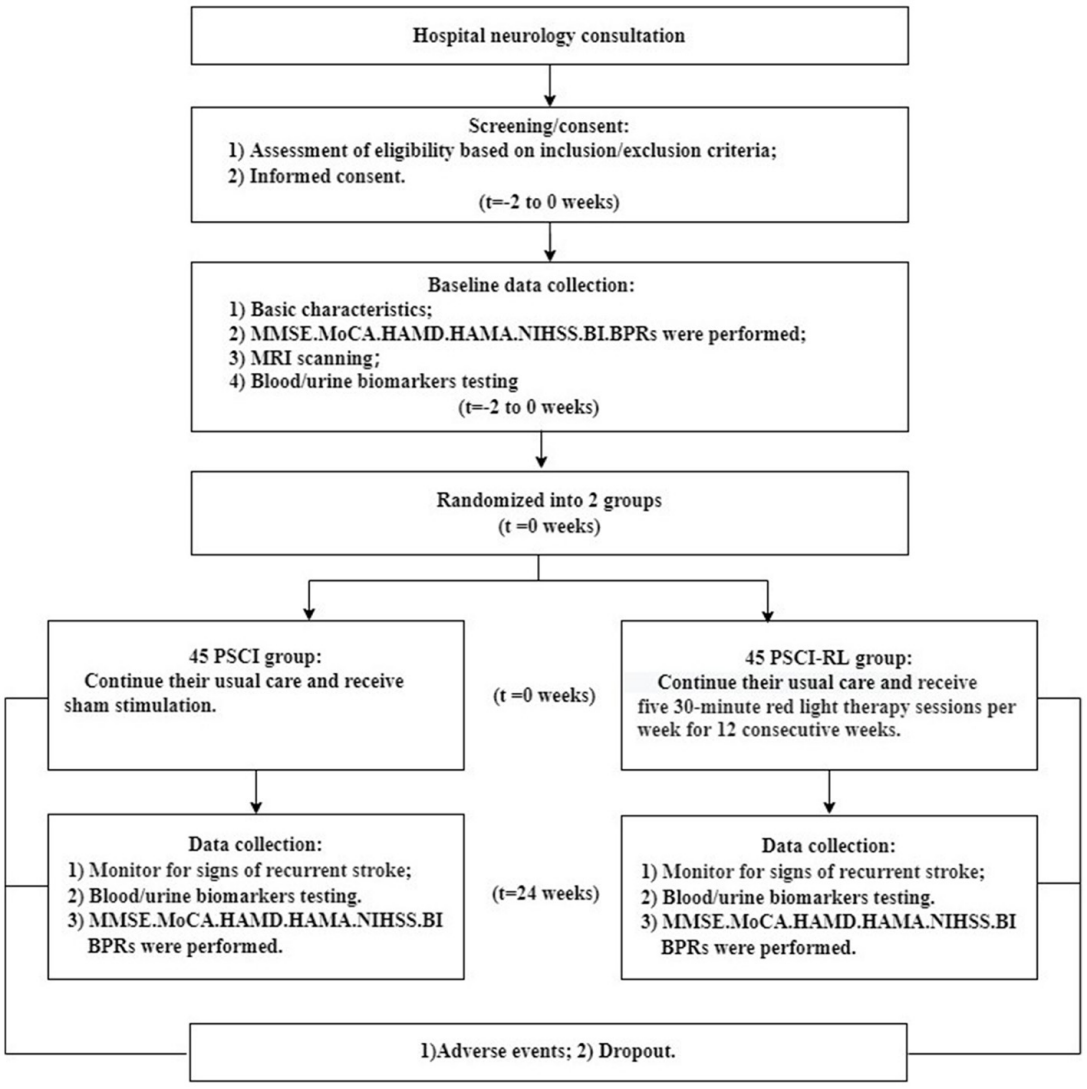
Flow diagram of study design.

The flow of study participants is shown in Figures 3, 4. A total of 90 patients were enrolled, with both groups receiving standard treatment for cerebrovascular diseases. Eligible patients were randomly assigned in a 1:1 ratio to either the PSCI-RL group (red light therapy) or the PSCI group (sham red light therapy). The randomization sequence was generated using a random number table, without stratification. Allocation was carried out by a designated investigator who was aware of the treatment assignments. To minimize bias, this study employed a single-blind design: patients and outcome assessors were blinded to the treatment allocation, while the interventionists were not blinded due to the practical constraints of administering the therapy. During the trial, one patient in the PSCI-RL group and seven patients in the PSCI group withdrew. Ultimately, 44 patients in the PSCI-RL group and 38 patients in the PSCI group completed the intervention and follow-up.

### 2.6 Demographic data collection

The demographic characteristics of all participants, encompassing age, gender, education level, body mass index, medical history, medication list, and comorbidities, were systematically documented in the case report form.

### 2.7 Blood and urine biomarkers

The 5 ml of fasting venous blood from subjects was drawn and centrifugation was performed to obtain the upper layer of plasma at 4 °C, 3,000 r, for 10 min. Urine was taken from the patient in the morning. Blood and urine biomarkers of all subjects were measured at baseline and 24 weeks. Detection of FA (NaFA), SSAO (YJ977822, mlbio, China), FDH (YJ141255, mlbio, China), Cyt-C (H190-1-2, Nanjing Jiancheng Bioengineering Insititute, China), CoQ10 (YJ403655, mlbio, China), and H_2_O_2_ (FT-P31666R, fantaibio.com, China) levels was carried out according to the instructions of the commercial kits, respectively.

### 2.8 Assessment of outcomes and safety assessment

The primary objective was to test the safety and effects of RL therapy on cognitive function, neuropsychiatric symptoms, and activities of daily living in patients with PSCI. The secondary objective was to: examine changes in the concentration or activity of blood biomarkers such as FA, FDH, SSAO, Cyt-c, and CoQ10. The patients were monitored from the time of enrollment until completion of the study for any adverse events defined in the Common Terms Criteria for Adverse Events (CTCAE) version 5.0. Reports of adverse events that might be related to the intervention were also available after the study. The safety of the new wearable brain rehabilitation device was assessed based on the incidence and severity of adverse events such as headache, dizziness, or nausea. Adverse events were reported immediately and managed by experienced physicians. Details of these possible adverse events and the treatment they received were recorded by completing a case report form. Physical examination and necessary laboratory tests such as routine blood tests, liver function, renal function, lipids, glycated hemoglobin, and thyroid function tests. were completed at the beginning of the study and the end of the study.

### 2.9 Statistical analysis

Statistical analyses were performed using IBM SPSS Statistics for Windows, Version 27.0 (IBM Corp., Armonk, NY, USA). Continuous variables were presented as mean ± standard deviation or 95% confidence intervals, and categorical variables as frequencies and percentages. Differences in baseline demographic and clinical characteristics between groups were assessed using independent-samples *t*-tests for continuous variables and Chi-square tests for categorical variables. The primary endpoint was analyzed under the intention-to-treat (ITT) principle. Missing data were assumed to be missing at random and were handled using the Multiple Imputation procedure in SPSS (Analyze → Multiple Imputation → Impute Missing Data), which generated five imputed datasets. All baseline variables and outcome variables were included in the imputation model to maintain internal associations. Each imputed dataset was analyzed separately, and pooled estimates were obtained automatically using Rubin's rules. Secondary and exploratory outcomes were analyzed in the per-protocol (PP) population without imputation. A two-sided *P* value < 0.05 was considered statistically significant.

## 3 Results

### 3.1 Demographic statistics of baseline

There were no significant differences between the two groups in terms of gender, age, body mass index, years of education, lipid profile, underlying diseases, history, and measurement of each scale as shown in the Table 1 (*P* > 0.05).

**Table 1:** Comparison of demographic characteristics and baseline assessments between the PSCI and PSCI-RL groups.

**Variables**	**PSCI *n* = 38**	**PSCI-RL *n* = 44**	***t*/χ^2^**	***P* value**
Age (y)	64.50 ± 8.35	61.14 ± 9.66	1.674	0.10
Sex (male)	26 (68.4)	31 (70.5)	0.040	0.84
BMI (kg/m^2^)	23.81 ± 3.40	23.88 ± 2.92	−0.104	0.92
Education (y)	5.18 ± 2.64	5.77 ± 4.31	−0.756	0.45
Diabetes	19 (50)	20 (45.5)	0.169	0.68
Hypertension	31 (81.6)	35 (79.5)	0.169	0.82
Hyperlipidemia	21 (56.8)	27 (61.4)	0.177	0.67
CHD	1 (2.6)	1 (2.3)	0.000	1.00
AF	2 (5.3)	6 (13.6)	0.812	0.37
Stroke history	7 (18.4)	3 (6.8)	1.595	0.21
Drinking history	13 (34.2)	18 (42.9)	0.628	0.43
Smoking history	16 (42.1)	14 (32.6)	0.788	0.38
Thrombolysis	3 (7.9)	8 (18.2)	1.858	0.17
Thrombectomy	0 (0)	2 (4.7)	0.395	0.53
TG (mmol/L)	1.63 ± 0.81	1.81 ± 1.24	−0.752	0.45
TC (mmol/L)	4.28 ± 0.98	4.43 ± 1.00	−0.728	0.47
HDL (mmol/L)	1.05 ± 0.28	1.02 ± 0.22	0.549	0.58
LDL (mmol/L)	2.67 ± 0.92	2.72 ± 0.97	−0.230	0.82
GHB (%)	6.65 ± 1.49	6.77 ± 1.93	−0.712	0.48
TSH (mIU/L)	1.94 ± 1.20	1.81 ± 1.38	0.442	0.66

AF, atrial fibrillation; BI, Barthel Index; BMI, body mass index; CHD, coronary heart disease; GHB, glycated hemoglobin; HAMA, Hamilton Anxiety Scale; HAMD, Hamilton Depression Scale; HDL, high-density lipoprotein; LDL, low-density lipoprotein; MMSE, mini-mental state examination; MoCA, Montreal cognitive assessment; NIHSS, National Institute of Health Stroke Scale; PSCI, post-stroke cognitive impairment; PSCI-RL, post-stroke cognitive impairment-red light; TC, total cholesterol; TG, triglyceride; TSH, thyroid stimulating hormone.

Categorical data are expressed as number of cases (percentage). The term “Stroke History” in our study specifically refers to previous cerebrovascular events excluding the current stroke being studied. “Drinking History” and “Smoking History” refer to a documented history of alcohol consumption and tobacco use, respectively, prior to the patient's enrollment. Regarding “Thrombolysis,” this refers only to patients who received thrombolytic treatment for the current stroke episode.

### 3.2 Changes in clinical outcomes after treatment

Pre-treatment scale scores did not differ significantly between groups (all *P* > 0.05), confirming baseline comparability. In the intention-to-treat (ITT) analysis, after 3 months of intervention both groups demonstrated significant within-group improvements in MMSE, MoCA, and BI scores, as well as significant reductions in HAMD, HAMA, NIHSS, and BPRS scores (all *P* < 0.05). Between-group comparisons demonstrated that the PSCI-RL group exhibited greater cognitive improvement than the control group, as reflected by higher post-treatment MMSE and MoCA scores. Specifically, the mean difference (MD) in MMSE was 3.36 (95% CI: 1.36–5.36), and the MD in MoCA was 3.48 (95% CI: 1.33–5.63), both favoring PSCI-RL. In addition, depressive symptoms improved more in the PSCI-RL group, with a HAMD mean difference of −1.53 (95% CI: −2.86 to −0.20). No statistically significant between-group differences were observed for HAMA, NIHSS, BI, or BPRS, with all 95% confidence intervals crossing zero, suggesting that improvement in these measures was similar between groups (Table 2). The per-protocol (PP) analysis produced a highly similar pattern of results, with consistent directions and levels of statistical significance, thereby supporting the robustness of the ITT findings (Supplementary Table S1).

**Table 2:** Comparison of cognitive and neuropsychiatric outcomes between groups (MD and 95% CI).

**Index**	**Treatment**	**PSCI (*n* = 45)**	**PSCI-RL (*n* = 45)**	**MD (95% CI)**	***t***	***P-*value**
MMSE score ($\bar{\text{x}}\pm \text{s}$)	Pre-treatment	18.53 ± 5.13	18.07 ± 6.37	3.36 (1.36, 5.36)	−0.383	0.703
	Post-treatment	20.22 ± 5.65^*^	23.58 ± 4.14^*^		3.213	0.002^#^
MoCA score ($\bar{\text{x}}\pm \text{s}$)	Pre-treatment	11.04 ± 4.50	12.07 ± 5.91	3.48 (1.33, 5.63)	0.923	0.359
	Post-treatment	13.46 ± 4.88^*^	16.94 ± 5.49^*^		3.178	0.002^#^
HAMD score ($\bar{\text{x}}\pm \text{s}$)	Pre-treatment	8.42 ± 3.73	9.31 ± 4.75	−1.53 (−2.86,−0.20)	0.987	0.326
	Post-treatment	6.39 ± 3.17^*^	4.86 ± 3.28^*^		−2.250	0.027^#^
HAMA score ($\bar{\text{x}}\pm \text{s}$)	Pre-treatment	9.73 ± 3.51	10.24 ± 4.48	−0.34 (−1.39, 0.71)	0.602	0.548
	Post-treatment	7.36 ± 2.24^*^	7.02 ± 2.79^*^		−0.642	0.523
NIHSS score ($\bar{\text{x}}\pm \text{s}$)	Pre-treatment	3.22 ± 1.72	3.51 ± 3.01	0.02 (−0.36, 0.40)	0.559	0.578
	Post-treatment	0.79 ± 0.84^*^	0.81 ± 0.98^*^		0.135	0.893
BI score ($\bar{\text{x}}\pm \text{s}$)	Pre-treatment	80.89 ± 17.53	78.78 ± 20.59	−1.71 (−5.75, 2.33)	−0.524	0.602
	Post-treatment	95.98 ± 8.59^*^	94.27 ± 10.83^*^		−0.829	0.409
BPRs score ($\bar{\text{x}}\pm \text{s}$)	Pre-treatment	22.38 ± 3.63	23.33 ± 4.51	−0.34 (−1.50, 0.82)	1.107	0.271
	Post-treatment	20.92 ± 3.07^*^	20.58 ± 2.52^*^		−0.558	0.578

^*^Denotes a difference significant at *P* < 0.05 when compared with pretest values.

^#^Denotes a difference significant at *P* < 0.05 when compared with the control group.

BI, Barthel Index; BPRs, brief psychiatric rating scale; HAMA, Hamilton Anxiety Scale; HAMD, Hamilton Depression Scale; MMSE, mini-mental state examination; MoCA, Montreal Cognitive Assessment; NIHSS, National Institute of Health Stroke Scale; PSCI, post-stroke cognitive impairment; PSCI-RL, post-stroke cognitive impairment-red light; MD, mean difference; CI, confidence interval.

### 3.3 Comparison of MoCA sub-item scores between groups

There was no significant difference in the MoCA scores of each sub-item between the two groups prior to RL treatment (*P* > 0.05). However, post-treatment scores for naming, attention, delayed recall, and orientation were significantly elevated (*P* < 0.05). Additionally, post-treatment scores for executive ability and language ability in the PSCI-RL group were significantly higher (*P* < 0.05). Patients in the PSCI-RL group demonstrated significantly higher scores in executive ability, abstraction ability, and orientation compared with the PSCI group (*P* < 0.05; Supplementary Table S2).

### 3.4 Biochemical index in the blood

**Figure 5:**
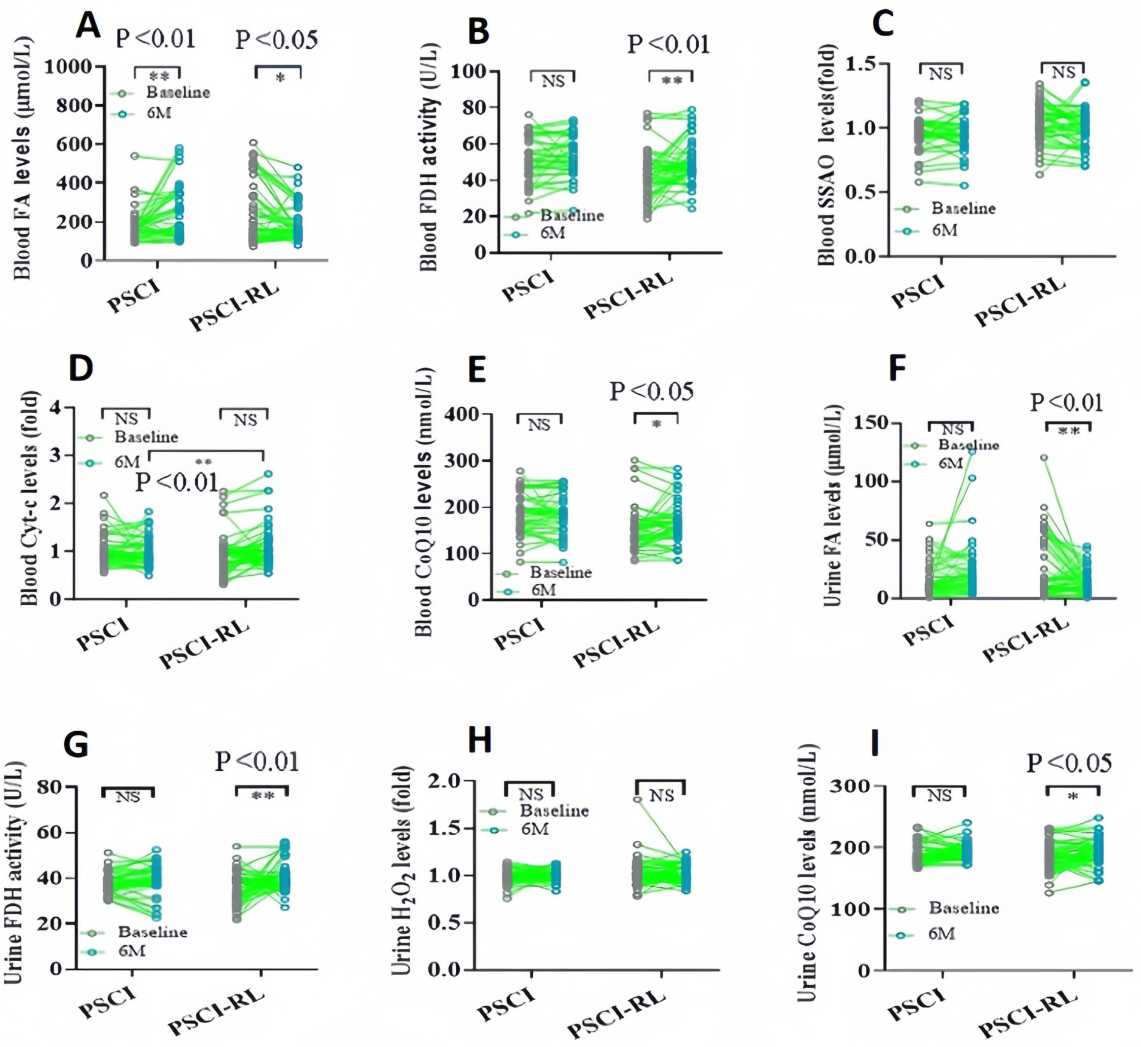
Longitudinal changes in biochemical indices in blood and urine from baseline to 6 months. **(A–E)** Blood FA, FDH, SSAO, Cyt-c, and CoQ10. **(F–I)** Urinary FA, FDH, H_2_O_2_, and CoQ10. *P < 0.05; **P < 0.01.

To evaluate changes in FA metabolism, blood FA levels were assessed using the Na-FA probe, a specific FA-sensitive sensor (36, 37). At 6 months, FA concentrations were significantly lower in the PSCI-RL group compared with the PSCI group (Figure 5A). These data indicate that RL therapy indeed enhances FA degradation in PSCI patients associated with cognitive improvement. Subsequently, we investigated alterations in FA-metabolic enzymes, specifically focusing on FDH and SSAO. At the 6-month assessment, FDH activity was significantly higher in the PSCI-RL group, whereas SSAO levels were reduced relative to the PSCI group (Figures 5B, C). These results suggest that 630-nm RL therapy activates FDH while concurrently inhibiting SSAO in PSCI patients. We further investigated Cyt-c, an enzyme that promotes CoQ10 generation, and FA can be scavenge by CoQ10 (38). Cyt-c levels were significantly elevated in the PSCI-RL group at 6 months compared with the PSCI group (Figure 5D). Consistently, CoQ10 concentrations were also significantly higher in the PSCI-RL group (Figure 5E). Together, these findings indicate that 630-nm RL therapy enhances FA metabolism by stimulating FDH activity, inhibiting SSAO, and promoting Cyt-c–mediated CoQ10 generation.

### 3.5 Biochemical index in the urine

Urinary biomarkers demonstrated a pattern consistent with blood measurements. At 6 months, urinary FA concentrations were significantly lower, and FDH activity was significantly higher in the PSCI-RL group compared with the PSCI group (Figures 5F, G). In addition, urinary H_2_O_2_ levels were reduced, while CoQ10 levels were elevated in the PSCI-RL group (Figures 5H, I). These findings further support the conclusion that RL therapy enhances systemic FA metabolism in PSCI patients.

### 3.6 Safety of RL therapy and its effects on stroke relapse

In this study, reports of adverse events potentially associated with the intervention were also accessible following the conclusion of the study. The safety profile of the novel wearable brain rehabilitation device was evaluated by examining the incidence and severity of adverse events, including headache, dizziness, and nausea. Adverse events were reported promptly and managed by experienced physicians. The safety monitoring indicated that no adverse events associated with the red-light therapy were reported in PSCI patients, nor were any documented by the investigating physicians. The intervention was well-tolerated.

In addition, we observed the relapse of stroke in both groups during the study period. The results of statistics showed that the relapse rate in the PSCI group (18.4%) was markedly higher than that in the PSCI-RL group (2.3%; *P* = 0.037; Table 3).

**Table 3:** Stroke relapse within 6 months in the PSCI and PSCI-RL groups [*n* (%)].

**Index**	**PSCI (*n* = 38)**	**PSCI-RL (*n* = 44)**	***Z*/χ^2^**	***P*-value**
Relapse within 6 months	7 (18.4)	1 (2.3)	4.344	0.037^*^

^*^Denotes *P* < 0.05.

PSCI, Post-Stroke Cognitive Impairment; PSCI-RL, Post-Stroke Cognitive Impairment-Red Light.

## 4 Discussion

In this exploratory study, patients received a 3-month intervention with 630-nm RL therapy. At the 6-month follow-up, the PSCI-RL group exhibited significantly higher MoCA and MMSE scores and markedly lower HAMD scores compared with the PSCI group. Biochemical analyses demonstrated that RL therapy significantly modulated FA metabolism–related parameters: FA levels in both blood and urine were notably reduced, accompanied by decreases in SSAO and its by-product H_2_O_2_, while FDH activity was significantly increased. Moreover, the levels of Cyt-c and CoQ10 in the treatment group were significantly higher than those observed in the PSCI group.

These therapeutic effects are consistent with an emerging body of evidence supporting the cognitive and neuropsychiatric benefits of PBM. Several studies have demonstrated that PBM can enhance cognitive function in various neurodegenerative conditions, such as traumatic brain injury (39, 40), Alzheimer's disease (41), and age-related cognitive impairment (42), while other research has highlighted its potential to relieve depressive symptoms (43). Notably, the consistency between the ITT and PP analyses (e.g., MoCA: MD = 3.66, 95% CI: 1.37–5.95 in PP analysis) further supports the robustness and stability of the observed treatment effects.

Furthermore, our study provides mechanistic insights into the observed therapeutic effects, suggesting that they may be mediated by the activation of FDH, which facilitates the degradation of FA (20). Supporting evidence includes increased FDH activity and CoQ10 levels, accompanied by decreases in SSAO and FA levels in PSCI patients. FA is a well-recognized gaseous environmental pollutant (44). However, endogenous FA is also present in all vertebrate cell as a byproduct of several metabolic reactions, such as methanol oxidation, and DNA or histone demethylation (45). Excessive FA exposure has been shown to cause memory decline in healthy adult mice (46–48), and FA accumulation in the brain is known to increase progressively with age, correlating with cognitive decline in humans (49). SSAO, expressed in vascular endothelium and circulating blood, can generate both FA and H_2_O_2_ (50). It has been found to have a strong relationship with stroke and vascular dementia (51), because its activity is positively correlated with intracranial hemorrhage in stroke patients (52), and dementia degree in patients with Alzheimer's disease (AD) (51, 53). SSAO is considered to be a potential target for stroke and AD (54). In contrast, FDH primarily functions to degrade FA (55). It has been shown that an imbalance in the expression and activity of SSAO and FDH is a key cause of FA accumulation during aging (19, 56). RL therapy has also been reported to enhance Cyt-c activity (57). Since excess FA inhibits Cyt-c activity in mitochondria and reduces CoQ10 levels, it ultimately induces neuronal death. However, CoQ10 as an endogenous FA scavenger can reduce FA levels in the brain (38). Taken together, these findings suggest that 630-nm RL therapy modulates FA metabolism through upregulation of FDH and downregulation of SSAO, thereby forming a coherent mechanistic pathway that may underlie the observed cognitive and emotional improvements in PSCI. Nevertheless, the precise upstream regulatory factors and downstream signaling cascades involved in this metabolic modulation remain to be fully elucidated, and further mechanistic studies are warranted.

In recent years, PBM has been established and used to improve cognitive dysfunction in brain diseases (58). RL (610–740 nm) and NIR (750–1,100 nm) can penetrate the human skull (59). Although the longer wavelengths of NIR in the 780–1,080 nm range have a stronger penetration ratio of skull than RL, the former also has a higher thermal effect on the brain than the latter (60). The wavelength of 630 nm used in this new device has less thermal effect than 680-nm RL (20). On the one hand, gaseous FA exposure can induce cognitive decline in animals (16) and humans (17). Injection of liquid FA directly promotes depression-like behaviors in animals (18). Hence, this new device of RL therapy could alleviate FA-related cognitive and mental disorders in PSCI patients. On the other hand, PBM has other mechanisms including an increase in cerebral blood flow (61) and an improvement in brain energy metabolism (62), but a decline in neuroinflammatory levels; thereby reducing the size of cerebral infarcts (63). In addition, PBM has a positive impact on muscle strength and mobility in stroke patients, improving their ability to perform activities of daily living (64). Thus, PBM is a promising and non-invasive approach for treating PSCI patients.

In addition, we observed a lower rate of stroke recurrence in the PSCI-RL group compared with the PSCI group during the follow-up period. Although the study was not originally powered or designed to evaluate recurrence as a primary endpoint and the sample size was relatively limited, this finding is noteworthy. One potential explanation is that improvements in cognitive function and emotional symptoms following RL therapy may facilitate better treatment adherence, more stable lifestyle patterns, and greater engagement in secondary prevention measures. Moreover, previous studies have shown that red-light photobiomodulation can promote vasodilation mediated by nitric oxide (NO) photorelease, enhance cerebral blood flow, and improve microvascular perfusion (65). RL therapy has also been reported to reduce neuroinflammatory responses, promote neuronal survival, and support synaptic repair following ischemic injury (66). These effects may collectively contribute to enhanced neurovascular stability and improved resilience to recurrent ischemic events.

However, given the exploratory nature of this study, caution is warranted when interpreting this finding. The sample size was modest, the follow-up period was limited, and unmeasured confounders—such as medication adherence, vascular risk factor control, and lifestyle behavior—may also influence recurrence outcomes. Therefore, this observation should be considered hypothesis-generating rather than conclusive. Future studies with larger cohorts, longer follow-up durations, and stratified recurrence-risk analyses are needed to determine whether RL therapy may have a stabilizing effect on cerebrovascular vulnerability or secondary prevention outcomes in PSCI patients.

This study has several limitations. First, a sham red-light control was not implemented due to practical and technical constraints. Although both groups used devices identical in appearance and treatment procedures, and all sessions were conducted individually to minimize perceptual awareness of group allocation, a slight thermal sensation induced by 630-nm RL cannot be entirely excluded. Future studies should refine the treatment apparatus by removing the electrotherapy component as an active co-intervention and incorporating a non-therapeutic light-emitting sham device with simulated heating, while adopting a double-blind design to further enhance methodological rigor. Second, the dropout rate was higher in the PSCI group than in the PSCI-RL group. Although most withdrawals were due to personal or family-related reasons and participants were blinded to group allocation, this imbalance may introduce bias and should be taken into account when interpreting the findings. Third, the follow-up duration was limited to 6 months; longer-term studies are required to evaluate the durability of treatment effects. Finally, although this exploratory clinical trial yielded encouraging preliminary outcomes, larger, multicenter randomized controlled studies with extended follow-up are needed to further validate the efficacy, safety, and generalizability of 630-nm RL therapy in PSCI.

## 5 Conclusions

In summary, this randomized controlled pilot study explored the potential benefits and safety of 630-nm RL therapy in patients with PSCI. The findings indicate that this novel PBM approach may offer a safe and non-invasive option for post-stroke cognitive and mental rehabilitation. However, given the exploratory design and limited sample size, the results should be interpreted with caution and require confirmation in larger, multicenter trials.

## Author's note

All the authors take responsibility for all aspects of the reliability and freedom from bias of the data presented and their discussed interpretation.

## Data Availability

The original contributions presented in the study are included in the article/Supplementary material, further inquiries can be directed to the corresponding authors.

## References

[1] Strong K Mathers C Bonita R. Preventing stroke: saving lives around the world. Lancet Neurol. (2007) 6:182–7. doi: 10.1016/S1474-4422(07)70031-5 17239805

[2] Mozaffarian D Benjamin EJ Go AS Arnett DK Blaha MJ Cushman M . Heart disease and stroke statistics-2016 update: a report from the American Heart Association. Circulation. (2016) 133:e38–360. doi: 10.1161/CIR.0000000000000350 26673558

[3] Ramos-Estebanez C Moral-Arce I Rojo F Gonzalez-Macias J Hernandez JL. Vascular cognitive impairment and dementia expenditures: 7-year inpatient cost description in community dwellers. Postgrad Med. (2012) 124:91–100. doi: 10.3810/pgm.2012.09.2597 23095429

[4] Huang YY Chen SD Leng XY Kuo K Wang ZT Cui M . Post-stroke cognitive impairment: epidemiology, risk factors, and management. J Alzheimers Dis. (2022) 86:983–99. doi: 10.3233/JAD-215644 35147548

[5] Leys D Hénon H Mackowiak-Cordoliani MA Pasquier F. Poststroke dementia. Lancet Neurol. (2005) 4:752–9. doi: 10.1016/S1474-4422(05)70221-0 16239182

[6] Iadecola C Duering M Hachinski V Joutel A Pendlebury ST Schneider JA . Vascular cognitive impairment and dementia: JACC scientific expert panel. J Am Coll Cardiol. (2019) 73:3326–44. doi: 10.1016/j.jacc.2019.04.034 31248555 PMC6719789

[7] Mijajlović MD Pavlović A Brainin M Heiss WD Quinn TJ Ihle-Hansen HB . Post-stroke dementia - a comprehensive review. BMC Med. (2017) 15:11. doi: 10.1186/s12916-017-0779-7 28095900 PMC5241961

[8] Delavaran H Jönsson AC Lövkvist H Iwarsson S Elmståhl S Norrving B . Cognitive function in stroke survivors: a 10-year follow-up study. Acta Neurol Scand. (2017) 136:187–94. doi: 10.1111/ane.12709 27804110

[9] Pendlebury ST Rothwell PM. Prevalence, incidence, and factors associated with pre-stroke and post-stroke dementia: a systematic review and meta-analysis. Lancet Neurol. (2009) 8:1006–18. doi: 10.1016/S1474-4422(09)70236-4 19782001

[10] Levine DA Galecki AT Langa KM Unverzagt FW Kabeto MU Giordani B . Trajectory of cognitive decline after incident stroke. JAMA. (2015) 314:41–51. doi: 10.1001/jama.2015.6968 26151265 PMC4655087

[11] Jia J Wei C Liang J Zhou A Zuo X Song H . The effects of DL-3-n-butylphthalide in patients with vascular cognitive impairment without dementia caused by subcortical ischemic small vessel disease: a multicentre, randomized, double-blind, placebo-controlled trial. Alzheimers Dement. (2016) 12:89–99. doi: 10.1016/j.jalz.2015.04.010 26086183

[12] Yin M Liu Y Zhang L Zheng H Peng L Ai Y . Effects of rTMS treatment on cognitive impairment and resting-state brain activity in stroke patients: a randomized clinical trial. Front Neural Circuits. (2020) 14:563777. doi: 10.3389/fncir.2020.563777 33117131 PMC7561423

[13] Gao Y Qiu Y Yang Q Tang S Gong J Fan H . Repetitive transcranial magnetic stimulation combined with cognitive training for cognitive function and activities of daily living in patients with post-stroke cognitive impairment: a systematic review and meta-analysis. Ageing Res Rev. (2023) 87:101919. doi: 10.1016/j.arr.2023.101919 37004840

[14] Chen X Liu F Lin S Yu L Lin R. Effects of virtual reality rehabilitation training on cognitive function and activities of daily living of patients with poststroke cognitive impairment: a systematic review and meta-analysis. Arch Phys Med Rehabil. (2022) 103:1422–35. doi: 10.1016/j.apmr.2022.03.012 35417757

[15] Hamblin MR Salehpour F. Photobiomodulation of the brain: shining light on Alzheimer's and other neuropathological diseases. J Alzheimers Dis. (2021) 83:1395–7. doi: 10.3233/JAD-210743 34459408

[16] Lu Z Li CM Qiao Y Yan Y Yang X. Effect of inhaled formaldehyde on learning and memory of mice. Indoor Air. (2008) 18:77–83. doi: 10.1111/j.1600-0668.2008.00524.x 18333987

[17] Kilburn KH R Warshaw Thornton JC. Formaldehyde impairs memory, equilibrium, and dexterity in histology technicians: effects which persist for days after exposure. Arch Environ Health. (1987) 42:117–20. doi: 10.1080/00039896.1987.9935806 3579365

[18] Zhao D Wu Y Zhao H Zhang F Wang J Liu Y . Midbrain FA initiates neuroinflammation and depression onset in both acute and chronic LPS-induced depressive model mice. Brain Behav Immun. (2024) 117:356–75. doi: 10.1016/j.bbi.2024.02.004 38320681

[19] Zhang J Yue X Luo H Jiang W Mei Y Ai L . Illumination with 630 nm red light reduces oxidative stress and restores memory by photo-activating catalase and formaldehyde dehydrogenase in SAMP8 mice. Antioxid Redox Signal. (2019) 30:1432–49. doi: 10.1089/ars.2018.7520 29869529

[20] Yue X Mei Y Zhang Y Tong Z Cui D Yang J . New insight into Alzheimer's disease: light reverses abeta-obstructed interstitial fluid flow and ameliorates memory decline in APP/PS1 mice. Alzheimers Dement. (2019) 5:671–84. doi: 10.1016/j.trci.2019.09.007 PMC6838540 31720368

[21] Lochhead JJ Ronaldson PT Davis TP. The role of oxidative stress in blood-brain barrier disruption during ischemic stroke: antioxidants in clinical trials. Biochem Pharmacol. 2024:116186. doi: 10.1016/j.bcp.2024.116186 38561092 PMC11410550

[22] Navarro-Gonzalez C Huerga-Gomez A Fazzari P. Nrg1 intracellular signaling is neuroprotective upon stroke. Oxid Med Cell Longev. (2019) 2019:3930186. doi: 10.1155/2019/3930186 31583038 PMC6754950

[23] Liu L Li Z Zhou H Duan W Huo X Xu W . Chinese Stroke Association guidelines for clinical management of ischaemic cerebrovascular diseases: executive summary and 2023 update. Stroke Vasc Neurol. (2023) 8:e3. doi: 10.1136/svn-2023-002998 38158224 PMC10800268

[24] Ding L Gu Z Chen H Wang P Song Y Zhang X . Phototherapy for age-related brain diseases: challenges, successes and future. Ageing Res Rev. (2024) 94:102183. doi: 10.1016/j.arr.2024.102183 38218465

[25] Katzman R Zhang MY Ouang Ya Q Wang ZY Liu WT Yu E . A Chinese version of the Mini-Mental State Examination; impact of illiteracy in a Shanghai dementia survey. J Clin Epidemiol. (1988) 41:971–8. doi: 10.1016/0895-4356(88)90034-0 3193141

[26] Nasreddine ZS Phillips NA Bédirian V Charbonneau S Whitehead V Collin I . The montreal cognitive assessment, MoCA: a brief screening tool for mild cognitive impairment. J Am Geriatr Soc. (2005) 53:695–9. doi: 10.1111/j.1532-5415.2005.53221.x 15817019

[27] Hamilton M. The assessment of anxiety states by rating. Br J Med Psychol. (1959) 32:50–5. doi: 10.1111/j.2044-8341.1959.tb00467.x 13638508

[28] Rodriguez-Seijas C Thompson JS Diehl JM Zimmerman M. A comparison of the dimensionality of the Hamilton Rating Scale for anxiety and the DSM-5 anxious-distress specifier interview. Psychiatry Res. (2020) 284:112788. doi: 10.1016/j.psychres.2020.112788 31978629

[29] Hamilton M. A rating scale for depression. J Neurol Neurosurg Psychiatry. (1960) 23:56–62. doi: 10.1136/jnnp.23.1.56 14399272 PMC495331

[30] Kwah LK Diong J. National Institutes of Health Stroke Scale (NIHSS). J Physiother. (2014) 60:61. doi: 10.1016/j.jphys.2013.12.012 24856948

[31] Mahoney FI Barthel DW. Functional evaluation. The Barthel Index. Md State Med J. (1965) 14:61–5. doi: 10.1037/t02366-000 14258950

[32] van Swieten JC Koudstaal PJ Visser MC Schouten HJ van Gijn J. Interobserver agreement for the assessment of handicap in stroke patients. Stroke. (1988) 19:604–7. doi: 10.1161/01.STR.19.5.604 3363593

[33] Lukoff D Liberman RP Nuechterlein KH. Symptom monitoring in the rehabilitation of schizophrenic patients. Schizophr Bull. (1986) 12:578–602. doi: 10.1093/schbul/12.4.578 3810065

[34] Bonzanino M Riolo M Battaglini I Perna M De Mattei M. PEALut in the dietary management of patients with acute ischemic stroke: a prospective randomized controlled clinical trial. J Clin Med. (2024) 13:509. doi: 10.3390/jcm13020509 38256644 PMC10816980

[35] Chow S-C Shao J Wang H Lokhnygina Y. Sample Size Calculations in Clinical Research. 3rd ed. New York, NY: CRC Press (2017).

[36] Ai L Tan T Tang Y Yang J Cui D Wang R . Endogenous formaldehyde is a memory-related molecule in mice and humans. Commun Biol. (2019) 2:446. doi: 10.1038/s42003-019-0694-x 31815201 PMC6884489

[37] Tang Y Zhao Y Lin W. Preparation of robust fluorescent probes for tracking endogenous formaldehyde in living cells and mouse tissue slices. Nat Protoc. (2020) 15:3499–526. doi: 10.1038/s41596-020-0384-7 32968251

[38] Fei X Zhang Y Mei Y Yue X Jiang W Ai L . Degradation of FA reduces Abeta neurotoxicity and Alzheimer-related phenotypes. Mol Psychiatry. (2021) 26:5578–91. doi: 10.1038/s41380-020-00929-7 33328587

[39] Naeser MA Saltmarche A Krengel MH Hamblin MR Knight JA. Improved cognitive function after transcranial, light-emitting diode treatments in chronic, traumatic brain injury: two case reports. Photomed Laser Surg. (2011) 29:351–8. doi: 10.1089/pho.2010.2814 21182447 PMC3104287

[40] Lee TL Chan DY Chan DT Cheung MC Shum DH Chan AS. Transcranial photobiomodulation improves cognitive function, post-concussion, and PTSD symptoms in mild traumatic brain injury. J Neurotrauma. (2025). doi: 10.1089/neu.2025.0048 40485299

[41] Guo R Li D Li F Ji L Liu H Qiao H . Effects of whole-head 810 nm near-infrared therapy on cognitive and neuropsychiatric symptoms in Alzheimer's disease: a pilot study. J Alzheimers Dis. (2025) 104:52–60. doi: 10.1177/13872877251313819 39910867

[42] Chan AS Lee TL Hamblin MR Cheung MC. Photobiomodulation enhances memory processing in older adults with mild cognitive impairment: a functional near-infrared spectroscopy study. J Alzheimers Dis. (2021) 83:1471–80. doi: 10.3233/JAD-201600 33998541

[43] Guu TW Cassano P Li WJ Tseng YH Ho WY Lin YT . Wearable, self-administered transcranial photobiomodulation for major depressive disorder and sleep: a randomized, double blind, sham-controlled trial. J Affect Disord. (2025) 372:635–42. doi: 10.1016/j.jad.2024.12.065 39706483

[44] Zhu L Jacob DJ Keutsch FN Mickley LJ Scheffe R Strum M . Formaldehyde (HCHO) as a hazardous air pollutant: mapping surface air concentrations from satellite and inferring cancer risks in the United States. Environ Sci Technol. (2017) 51:5650–7. doi: 10.1021/acs.est.7b01356 28441488

[45] Kalasz H. Biological role of formaldehyde, and cycles related to methylation, demethylation, and formaldehyde production. Mini Rev Med Chem. (2003) 3:175–92. doi: 10.2174/1389557033488187 12570834

[46] Tong Z Han C Luo W Wang X Li H Luo H . Accumulated hippocampal formaldehyde induces age-dependent memory decline. Age. (2013) 35:583–96. doi: 10.1007/s11357-012-9388-8 22382760 PMC3636394

[47] Tong Z Wang W Luo W Lv J Li H Luo H . Urine formaldehyde predicts cognitive impairment in post-stroke dementia and Alzheimer's disease. J Alzheimers Dis. (2017) 55:1031–8. doi: 10.3233/JAD-160357 27802225

[48] Wang F Chen D Wu P Klein C Jin C. Formaldehyde, epigenetics, and Alzheimer's disease. Chem Res Toxicol. (2019) 32:820–30. doi: 10.1021/acs.chemrestox.9b00090 30964647 PMC6878761

[49] Yu J Su T Zhou T He Y Lu J Li J . Uric formaldehyde levels are negatively correlated with cognitive abilities in healthy older adults. Neurosci Bull. (2014) 30:172–84. doi: 10.1007/s12264-013-1416-x 24733650 PMC5562661

[50] Jakobsson E Nilsson J Ogg D Kleywegt GJ. Structure of human semicarbazide-sensitive amine oxidase/vascular adhesion protein-1. Acta Crystallogr D Biol Crystallogr. (2005) 61(Pt 11):1550–62. doi: 10.1107/S0907444905028805 16239734

[51] Yu PH. Involvement of cerebrovascular semicarbazide-sensitive amine oxidase in the pathogenesis of Alzheimer's disease and vascular dementia. Med Hypotheses. (2001) 57:175–9. doi: 10.1054/mehy.2001.1329 11461168

[52] Hernandez-Guillamon M Garcia-Bonilla L Solé M Sosti V Parés M Campos M . Plasma VAP-1/SSAO activity predicts intracranial hemorrhages and adverse neurological outcome after tissue plasminogen activator treatment in stroke. Stroke. (2010) 41:1528–35. doi: 10.1161/STROKEAHA.110.584623 20538694

[53] Yu PH Wright S Fan EH Lun ZR Gubisne-Harberle D. Physiological and pathological implications of semicarbazide-sensitive amine oxidase. Biochim Biophys Acta. (2003) 1647:193–9. doi: 10.1016/S1570-9639(03)00101-8 12686132

[54] Unzeta M Hernàndez-Guillamon M Sun P Solé M. SSAO/VAP-1 in cerebrovascular disorders: a potential therapeutic target for stroke and Alzheimer's disease. Int J Mol Sci. (2021) 22:3365. doi: 10.3390/ijms22073365 33805974 PMC8036996

[55] Teng S Beard K Pourahmad J Moridani M Easson E Poon R . The formaldehyde metabolic detoxification enzyme systems and molecular cytotoxic mechanism in isolated rat hepatocytes. Chem Biol Interact. (2001) 130–132:285–96. doi: 10.1016/S0009-2797(00)00272-6 11306052

[56] Qiang M Xiao R Su T Wu BB Tong ZQ Liu Y . A novel mechanism for endogenous formaldehyde elevation in SAMP8 mouse. J Alzheimers Dis. (2014) 40:1039–53. doi: 10.3233/JAD-131595 24583407

[57] Uozumi Y Nawashiro H Sato S Kawauchi S Shima K Kikuchi M. Targeted increase in cerebral blood flow by transcranial near-infrared laser irradiation. Lasers Surg Med. (2010) 42:566–76. doi: 10.1002/lsm.20938 20662034

[58] de la Torre JC. Treating cognitive impairment with transcranial low level laser therapy. J Photochem Photobiol B. (2017) 168:149–55. doi: 10.1016/j.jphotobiol.2017.02.008 28219828

[59] Firbank M Hiraoka M Essenpreis M Delpy DT. Measurement of the optical properties of the skull in the wavelength range 650-950 nm. Phys Med Biol. (1993) 38:503–10. doi: 10.1088/0031-9155/38/4/002 8488176

[60] Henderson TA Morries LD. Near-infrared photonic energy penetration: can infrared phototherapy effectively reach the human brain? Neuropsychiatr Dis Treat. (2015) 11:2191–208. doi: 10.2147/NDT.S78182 26346298 PMC4552256

[61] Rojas JC Bruchey AK Gonzalez-Lima F. Low-level light therapy improves cortical metabolic capacity and memory retention. J Alzheimers Dis. (2012) 32:741–52. doi: 10.3233/JAD-2012-120817 22850314

[62] Purushothuman S Johnstone DM Nandasena C Mitrofanis J Stone J. Photobiomodulation with near infrared light mitigates Alzheimer's disease-related pathology in cerebral cortex - evidence from two transgenic mouse models. Alzheimers Res Ther. (2014) 6:2. doi: 10.1186/alzrt232 24387311 PMC3978916

[63] Vogel DDS Ortiz-Villatoro NN Araújo NS Marques MJG Aimbire F Scorza FA . Transcranial low-level laser therapy in an *in vivo* model of stroke: Relevance to the brain infarct, microglia activation and neuroinflammation. J Biophotonics. (2021) 14:e202000500. doi: 10.1002/jbio.202000500 33580734

[64] de Jesus Fonseca EG Pedroso A Neuls D Barbosa D Cidral-Filho FJ Salgado ASI . Study of transcranial therapy 904 nm in experimental model of stroke. Lasers Med Sci. (2019) 34:1619–25. doi: 10.1007/s10103-019-02758-9 30826952

[65] Li Y Zhang L Lin J Yang L Duan R. Photobiomodulation in stroke prevention and treatment: neuroprotective mechanisms and therapeutic challenges. Brain Res. (2025) 1868:149981. doi: 10.1016/j.brainres.2025.149981 41067675

[66] Nairuz T. Sangwoo-Cho, Lee J-H. Photobiomodulation therapy on brain: pioneering an innovative approach to revolutionize cognitive dynamics. Cells. (2024) 13:966. doi: 10.3390/cells13110966 38891098 PMC11171912

