# Supplementary material for: Red-light photobiomodulation improves cognition and neuropsychiatric symptoms in post-stroke cognitive impairment: a randomized trial
Source: Front Neurol. 2025 Nov 26;16:1634701. doi: 10.3389/fneur.2025.1634701 (PMC12689328; doi:10.3389/fneur.2025.1634701)
Supplement: Supplementary file 1 [file Table_1.docx]

**Appendices**

**Table S1.** Comparison of MMSE, MoCA, HAMD, HAMA, NIHSS, BI scores between the PSCI and PSCI-RL groups (MD and 95% CI) .

| **Index** | **Treatment** | **PSCI**  **(n=38)** | **PSCI-RL**  **(n=44)** | **MD (95% CI)** | **t** | **P** |
| --- | --- | --- | --- | --- | --- | --- |
| **MMSE score**  **(x̅±s)** | Pre-treatment  Post-treatment | 18.47±5.43  20.74±5.17* | 18.07±6.44  23.52±4.17* | 2.78(0.73, 4.83) | -0.305  2.699 | 0. 761  0.008^#^ |
| **MoCA score**  **(x̅±s)** | Pre-treatment  Post-treatment | 11.26±4.60  13.18±5.08* | 12.09±5.98  16.84±5.51* | 3.66(1.37, 5.95) | 0.695  3.107 | 0.489  0.003^#^ |
| **HAMD score**  **(x̅±s)** | Pre-treatment  Post-treatment | 8.66±3.94  6.47±2.55* | 9.41±4.88  4.84±3.31* | −1.63(−2.90, −0.36) | 0.759  -2.469 | 0.450  0.016^#^ |
| **HAMA score**  **(x̅±s)** | Pre-treatment  Post-treatment | 9.79±3.66  7.55±2.11* | 10.18±4.51  7.05±2.81* | −0.50(−1.57, 0.57) | 0.428  -0.911 | 0.670  0.365 |
| **NIHSS score**  **(x̅±s)** | Pre-treatment  Post-treatment | 3.13±1.38  0.92±0.71* | 3.45±2.31  0.98±1.05* | 0.06(−0.32, 0.44) | 0.754  0.280 | 0.453  0.780 |
| **BI score**  **(x̅±s)** | Pre-treatment  Post-treatment | 82.37±14.97  97.63±6.44* | 79.20±20.175  94.66±10.80* | −2.97(−6.76, 0.82) | -0.796  -1.483 | 0.429  0.142 |
| **BPRs score**  **(x̅±s)** | Pre-treatment  Post-treatment | 22.53±3.82  21.26±3.13* | 23.30±4.55  20.47±2.45* | −0.79(−2.02, 0.44) | -0.821  -1. 109 | 0.414  0.278 |

*Denotes a difference significant at p < 0.05 when compared with pretest values; **^#^**denotes a difference significant at p < 0.05 when compared with the control group.

***Abbreviation:*** BI, Barthel Index; BPRs, Brief Psychiatric Rating Scale; HAMA, Hamilton Anxiety Scale; HAMD, Hamilton Depression Scale; MMSE, Mini-Mental State Examination; MoCA, Montreal Cognitive Assessment; NIHSS, National Institute of Health Stroke Scale; PSCI, Post-Stroke Cognitive Impairment; PSCI-RL, Post-Stroke Cognitive Impairment-Red Light; MD = mean difference; CI = confidence interval.

**
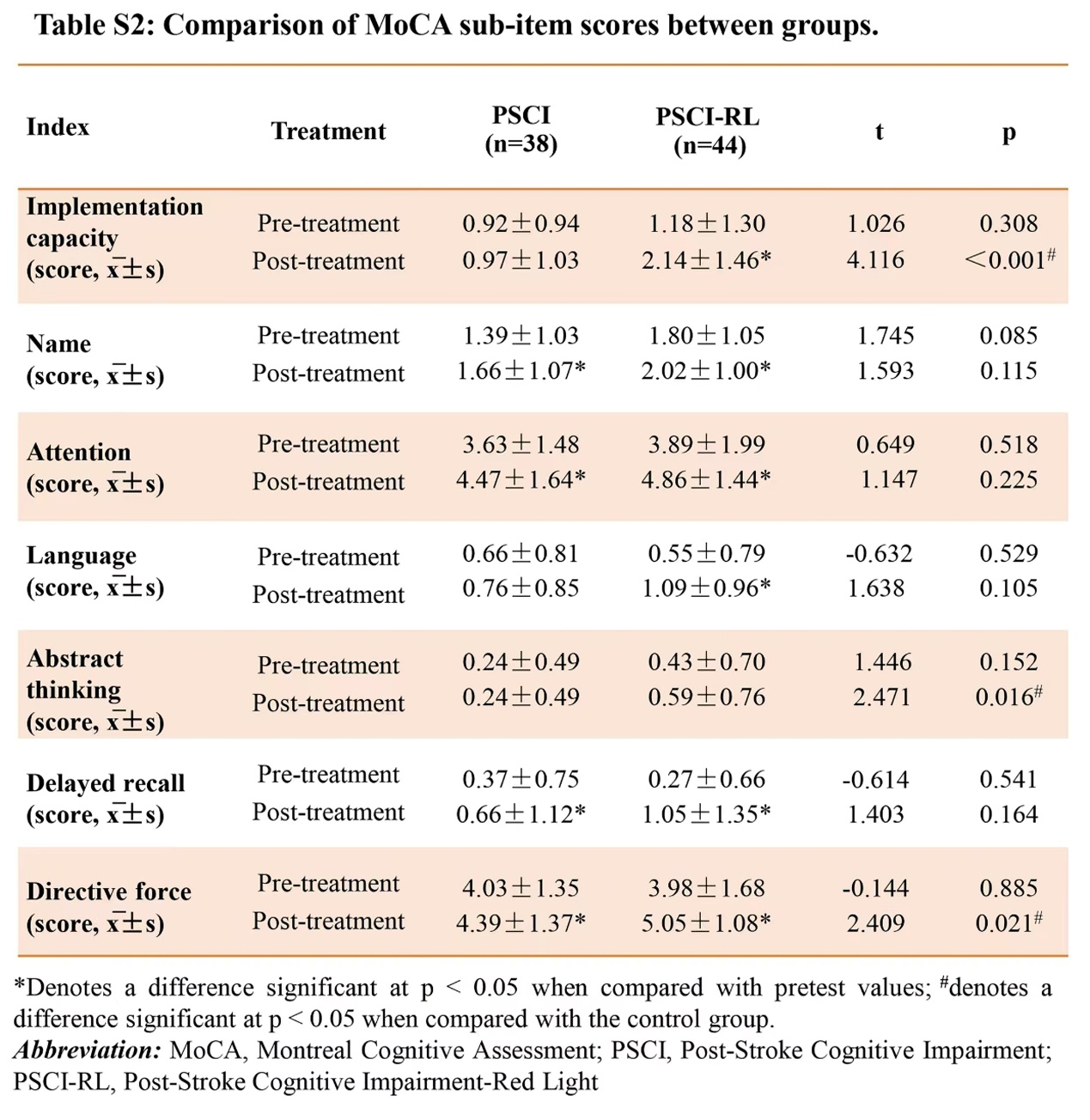
**
